# Assessment of Long-Term Changes in Knowledge and Attitudes of Household Contacts of COVID-19 Cases in Northern Spain

**DOI:** 10.3390/idr16050074

**Published:** 2024-09-23

**Authors:** Noelia Vera-Punzano, Vanessa Bullón-Vela, Carme Miret, Jéssica Pardos-Plaza, Manuel García Cenoz, Pere Godoy, Jesús Castilla, Àngela Domínguez, Diana Toledo, Iván Martínez-Baz

**Affiliations:** 1 Instituto de Salud Pública de Navarra, 31003 Pamplona, Spain; 2 Instituto de Investigación Sanitaria de Navarra (IdiSNA), 31008 Pamplona, Spain; 3 Institut de Recerca Biomédica (IRB Lleida), Universitat de Lleida, 25006 Lleida, Spain; 4 Agència de Salut Pública de Catalunya, 08005 Barcelona, Spain; 5 CIBER Epidemiología y Salud Pública (CIBERESP), Instituto de Salud Carlos III, 28029 Madrid, Spain; 6 Departament de Medicina, Universitat de Barcelona, 08036 Barcelona, Spain; 7 Fundació Bosch I Gimpera, 08028 Barcelona, Spain

**Keywords:** knowledge, attitude, COVID-19, household contact, preventive measures

## Abstract

This study aims to describe the long-term changes in the knowledge of, and attitudes towards, COVID-19 and its preventive measures in northern Spain. A telephonic survey was performed among household contacts of COVID-19 cases in Catalonia and Navarre between May 2022 and December 2023. Knowledge and attitudes were assessed through 12 questions using a Likert scale, and responses were grouped as correct or incorrect. The change from baseline to the 6-month follow-up was evaluated with the absolute difference (AD) using the proportion of correct answers. At baseline, 299 subjects were contacted, of whom 63.2% (189) completed the 6-month follow-up. Correct knowledge of transmission (>85%) and the use of preventive measures (>92%) were observed at baseline and maintained over time. The attitudes towards face mask use remained adequate over the course of six months (>79%). However, attitudes regarding the use of face masks indoors (AD = −16.4%; *p* < 0.001) and those who thought that COVID-19 had a negative impact on their lives (AD = −16.5%; *p* < 0.001) decreased after 6 months. In the post-acute phase of the pandemic, household contacts maintained the correct level of knowledge towards COVID-19, while some attitudes decreased. These results should serve as a guide for health policy makers in decision-making in case of a new increase in the incidence of SARS-CoV-2.

## 1. Introduction

COVID-19 is a highly infectious disease caused by SARS-CoV-2, which was officially declared as a public health emergency of international concern by the World Health Organization on 30 January 2020 [1,2,3]. There have been heavy social repercussions since it is spread by human-to-human transmission through droplets, respiratory secretions or direct contact [2,3].

At the beginning of the pandemic, with a lack of vaccines or effective antiviral therapies, the implementation of non-pharmaceutical preventive measures became essential to limit COVID-19 transmission. Non-pharmaceutical preventive measures include frequent handwashing, face mask use and social distancing [2,3,4]. The preventive measures were implemented but their intensity varied throughout the different phases of the pandemic, with their effectiveness limited by short-term compliance by society [5,6]. A study analyzing the effectiveness of non-pharmaceutical interventions from September 2020 to May 2021 concluded that increasing restrictions had a considerable effect in decreasing COVID-19 transmission [7].

Knowledge, attitudes and preventive practices play essential roles in SARS-CoV-2 containment, as per a model that highlights knowledge as the basis and attitude as the impulse of behavioural changes [8]. However, as time passes and the incidence of SARS-CoV-2 declines, the perception of the risk could decrease, and with it, the knowledge of and attitudes towards non-pharmaceutical preventive measures. Therefore, it is important to understand citizens’ knowledge and attitudes regarding COVID-19 and its preventive measures over time to identify the needs of the population and to adopt effective response measures in case of a new increase in the incidence of SARS-CoV-2 infections [9].

In this context, this study aims to describe long-term changes in knowledge and attitudes towards COVID-19 and its preventive measures among household contacts in northern Spain.

## 2. Materials and Methods

### 2.1. Study Design and Participants

A prospective epidemiological study was performed through a telephone survey of adult household contacts (≥18 years) of confirmed COVID-19 cases. Through epidemiological surveillance units in Navarre and Catalonia (northern Spain), eight primary healthcare centres, representative of each region, were selected to recruit household contacts between May 2022 and December 2023.

The methodology used in the study has been described previously [10]. In brief, a systematic recruitment of COVID-19 cases was conducted, and they were interviewed to identify their household contacts, who were invited to participate in the study. Household contacts were defined as permanent residents of the same address as the COVID-19 index case and individuals who had at least two hours of contact with the index case at their place of residence during the transmission period of SARS-CoV-2. The exclusion criteria were having a cognitive or hearing impairment that could prevent them from completing the surveys and being under 18 years of age.

### 2.2. Questionnaire

The questionnaire used and its validation have been described previously [10]. The questionnaire was developed and adapted from previously validated questionnaires and incorporated the recommendations of health institutions at national and international level [10,11]. A pilot test was performed prior to the study to ensure the viability and reliability of the questionnaire. The questionnaire consisted of seven sections, and trained personnel performed the telephone survey of household contacts in three rounds: baseline, after identification as a household contact, and 3 and 6 months later.

**Figure 1:**
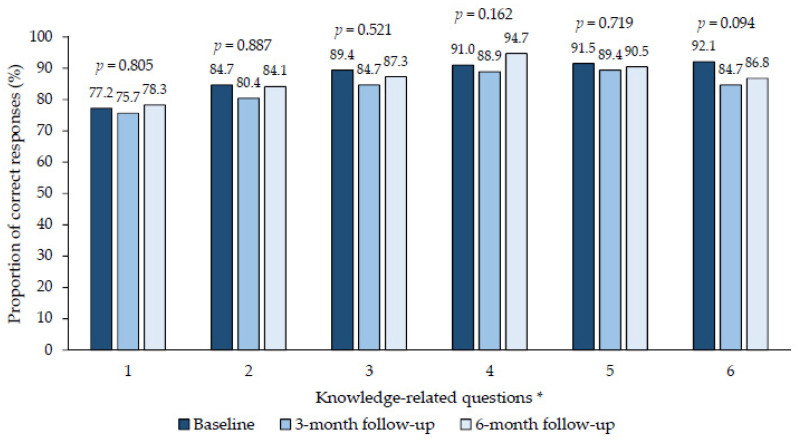
Long-term changes in the knowledge of COVID-19 disease and its preventive measures. * 1: All people who become ill with COVID-19 will develop into a severe case. 2: Asymptomatic persons diagnosed with COVID-19 can transmit the infection. 3: Persons diagnosed with COVID-19 can transmit infection despite vaccination. 4: The SARS-CoV-2 virus is spread by respiratory droplets from infected individuals when coughing/sneezing/talking/laughing/singing. 5: Handwashing is important to reduce the risk of contracting COVID-19. 6: To prevent transmission of COVID-19, measures such as wearing face masks and avoiding crowds in enclosed spaces should be maintained. Note: in question one, “totally disagree” or “disagree” were considered correct, and in the remaining five questions, “agree” or “totally agree” were considered correct.

**Figure 2:**
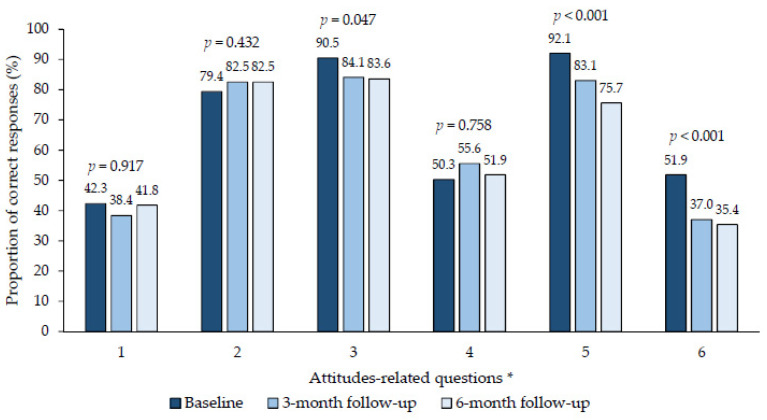
Long-term changes in attitudes about COVID-19 disease and its preventive measures. * 1: I consider myself susceptible to developing a severe disease if I become ill with COVID-19. 2: I consider that my inner circle has complied with the preventive measures to avoid becoming ill with COVID-19. 3: I consider that my inner circle has complied with the vaccination recommendations given by the health authorities. 4: I consider that it is better to develop immunity by getting sick with COVID-19 than by being vaccinated. 5: It is convenient for the general population to wear a face mask correctly (covering the nose and mouth) to prevent COVID-19 in crowded closed environments. 6: I consider that COVID-19 had a negative influence on my daily life. Note: in question four, “totally disagree” or “disagree” were considered an adequate attitude; in the remaining questions, “agree” or “totally agree” were considered adequate protective behaviour, except for question one, in which “agree” or “totally agree” were considered adequate protective behaviours if the participant had a major chronic condition, and inadequate if the participant did not have any chronic diseases.

The first part of the telephone survey consisted of four sections which obtained the patient’s sociodemographic and epidemiological information, and this information was obtained only at the baseline round. The second part, made out of three sections, focused on the patient’s knowledge of, and attitudes towards COVID-19 and its preventive measures. These were evaluated through 6 questions each and assessed using a 5-point Likert scale (totally agree, agree, neither agree nor disagree, disagree and totally disagree) [11]. At baseline, the questions about knowledge and attitudes were evaluated and repeated in the 3- and 6-month follow-up interviews to measure changes in the outcomes over time. The outcomes were the responses grouped into two categories—correct or adequate (agree or totally agree) and incorrect or inadequate (neither agree nor disagree, disagree or totally disagree)—with some exceptions specified in the Figure 1 and Figure 2.

### 2.3. Statistical Analyses

For this study, we included information from all rounds to describe the evolution in the knowledge and attitudes of participants over time. A descriptive analysis was carried out to evaluate the proportion of household contacts who responded correctly in each of the three rounds. The proportion of household contacts who responded correctly to the knowledge and attitude questions at baseline and at the 6-month follow-up was compared using the chi-square test, and possible changes between these rounds were assessed through the absolute difference (AD). Stratified analysis by age group (<60 and ≥60 years old), educational level (primary/secondary vs. higher educational level), the presence of high-risk conditions for severe COVID-19, and prior SARS-CoV-2 infection were performed. *p*-values lower than 0.05 were considered statistically significant.

### 2.4. Ethical Considerations

The Bioethics Commission of the University of Barcelona approved the study protocol (IRB00003099, approved on 02 March 2022) and all participants provided oral consent to participate in the study.

## 3. Results

### 3.1. Characteristics of Participants

A total of 299 household contacts were identified during the study period, of which 63% (*n* = 189) were eligible and completed the questionnaire for all three rounds (baseline and 3-month and 6-month follow-up). Of the participants included in the study, 63.0% (*n* = 119) of them were <60 years old, 53.4% (*n* = 101) were males, and 47.6% (*n* = 90) had a high educational level. Furthermore, 60.3% (*n* = 114) of participants presented with at least one risk factor for severe COVID-19, and 53.4% (*n* = 101) had a previous SARS-CoV-2 infection.

### 3.2. Long-Term Changes in Knowledge towards COVID-19 and Its Preventive Measures

Overall, no significant changes in knowledge were observed in the long term (Figure 1). Participants maintained the correct level of knowledge in both rounds (from baseline to the 6-month follow-up) about COVID-19 transmission from the asymptomatic (AD= −0.6; 84.7% vs. 84.1%; *p* = 0.887) and vaccinated population (AD= −2.1; 89.4% vs. 87.3%; *p* = 0.521).

In addition, at the 6-month follow-up, participants showed high awareness of non-pharmaceutical preventive measures such as the importance of handwashing (AD = −1.0, 91.5% vs. 90.5%; *p* = 0.719), or wearing face masks and avoiding crowds in enclosed spaces (AD= −5.3; 92.1% vs. 86.8%; *p* = 0.094).

The question relating to “not all people who become ill with COVID-19 will develop into a severe case ” was also stable over time (AD = 1.1; 77.2% vs. 78.3%; *p* = 0.805). The proportion of respondents who correctly answered that the COVID-19 virus is spread by respiratory droplets from infected individuals increased by 3.7 percentage points (91.0% vs. 94.7%; *p* = 0.162) over six months, although these differences were not statistically significant.

In the stratified analysis, similar results on knowledge in the long term were observed. However, among household contacts who had a previous SARS-CoV-2 infection or were aged 60 years or older, a significant decrease over time for the question “to prevent transmission of COVID-19, measures such as wearing face masks and avoiding crowds in enclosed spaces should be maintained” was observed in both subgroups (AD= –8.9; 93.1% vs. 84.2%; *p* = 0.046 and AD= –14.2; 97.1% vs. 82.9%; *p* = 0.005, respectively) over six months (Table 1).

### 3.3. Long-Term Changes in Attitudes towards COVID-19 and Its Preventive Measures

Figure 2 shows overall changes in attitudes over the follow-up period. The attitudes relating to the preventive measures that were followed by their inner circle (AD = 3.1; 79.4% vs. 82.5%; *p* = 0.432), self-awareness of being at risk of severe COVID-19 (AD = −0.5; 42.3% vs. 41.8%; *p* = 0.917), and the immunity developed by being vaccinated (AD = 1.6; 50.3% vs. 51.9%; *p* = 0.758) were maintained at the 6-month follow-up.

However, a significant long-term decrease was observed in the attitudes of participants relating to the compliance of their inner circle to the vaccination recommendations (AD = −6.9; 90.5% vs. 83.6%; *p* = 0.047), preventive measures in crowded closed environments (AD = −16.4; 92.1% vs. 75.7%; *p* < 0.001), and the negative influence of COVID-19 on their daily lives (AD = −16.5; 51.9% vs. 35.4%; *p* < 0.001) over six months.

All stratified analyses by age group, educational level, the presence of high risk factors, and previous infection showed a significant decrease over time in the attitudes related to the preventive measures in crowded closed environments, with a range of −12.5% to −19.8% in the AD in the long term. The proportion of participants who considered that COVID-19 had a negative influence on their daily lives decreased over time for almost all subgroups studied (AD range between −14.0% and −23.3%). However, the only group that experienced a significant decrease when considering whether their inner circle had complied with the preventive measures was that containing subjects with a higher educational level (AD = −13.4; *p* = 0.022) (Table 2).

## 4. Discussion

The present study shows the correct level of knowledge and adequate attitudes in household contacts of confirmed COVID-19 cases towards the infection and its preventive measures between 2022 and 2023 in northern Spain. The correct level of knowledge was generally maintained over time. However, in specific populations, a long-term change in some attitudes was observed, mainly in younger adults, people with a higher level of education, and those with no previous infection.

Correct knowledge of transmission (>85%) and preventive measures (>92%) were observed in the first round and remained stable after six months when the third round was conducted. These findings match the results of the COSMO-SPAIN study obtained for the 12th and last round, conducted in September 2022, with 70–95% correct statements for items related to knowledge of COVID-19 transmission and prevention [12]. However, although it was still high overall, the knowledge about COVID-19 in that study declined compared to previous rounds. Such behaviour was not observed in our evaluation of either the 3- [11] or 6-month follow-up.

This study was conducted in the post-acute phase of the COVID-19 pandemic in Spain, a period characterized by the relaxation of non-pharmaceutical interventions to prevent its transmission and the end of COVID-19 as a public health emergency of international concern. In Spain, the last mandatory non-pharmaceutical preventive measure to be withdrawn was the use of face masks in certain vulnerable enclosed spaces (e.g., pharmacies), which ceased in July 2023 [13]. After that, we entered a period in which self-awareness and social responsibility were the main guidelines to contain the circulation of COVID-19 in our environment. Public health policies during the study period showed no short-term changes in the level of knowledge and attitudes [11], but these interventions may have led to a decline in the level of adequate attitudes in the population in the long term.

In general, attitudes of household contacts towards preventive measures were adequate (>79%). However, attitudes regarding the use of face masks indoors and those who considered that COVID-19 had a negative impact on their lives decreased after a 6-month follow-up. Among the studied subgroups, younger adults (<60 years old), those with a higher level of education, and individuals with no previous infection had the greatest decrease in those attitudes. Other studies conducted in earlier stages of the COVID-19 pandemic also concluded that younger groups had the lowest knowledge and attitude, while they found that participants with a higher educational level were more likely to have better knowledge and attitudes [14,15]. The educational level may have increased in younger adults, with 80% of adults younger than 60 having a higher level of education in this study. However, different significant long-term changes in correct knowledge and adequate attitudes were observed depending on the age group and educational level.

A previous study using the same methodology as the present one, evaluated short-term changes in these items and concluded that the level of attitudes was adequate, with no significant changes observed over a 3-month period [11]. Conversely, as seen in the present study, when the period is extended to six months, some attitudes decrease significantly. Similar trends were observed in the COSMO-SPAIN study in September 2022 [12]. A study conducted in India during the first waves of the COVID-19 pandemic revealed a negative change in the attitude of the respondents despite the rise in knowledge [16]. This means that, even in the acute phase of the pandemic, the level of adequate attitudes did not match the knowledge.

The post-acute phase of the pandemic is influenced by a factor known as “pandemic fatigue”. This feeling was defined by the WHO as an individual’s feeling of demotivation to follow recommended protective behaviours, emerging gradually over time and potentially influenced by the relaxation of non-pharmaceutical preventive measures [17]. A Spanish study described changes in knowledge, attitude and practices between pandemic rounds, concluding that the significant decrease in the level of concern observed over time was greater in those with high “pandemic fatigue” [18]. This may be a contributing factor for the significant decline in positive attitudes. This was mainly among younger adults as this population tends to acquire and accept new information more quickly, meaning their response may change as public health policies evolve.

In the interpretation of these results, some limitations should be considered. This study included participants from two regions in northern Spain, with the same indications for preventive measures. However, the results may not be generalizable to other countries, in which similar indications for non-pharmaceutical preventive measures have not been carried out throughout the territory. The low statistical power in some stratified analyses, due to the low incidence of COVID-19 cases in the post-acute phase of the pandemic included in the study period, implies that they should be interpreted cautiously. The study used a telephone survey method and the information obtained was self-reported, so information bias may have been introduced in the outcome variables. However, this information was verified with electronic medical records, reducing the information bias. Changes in restrictions and the low incidence of SARS-CoV-2 infection may have negatively affected the assessment of long-term changes in knowledge and attitudes about COVID-19 and the non-pharmaceutical preventive measures.

## 5. Conclusions

In conclusion, household contacts of COVID-19 cases showed a correct level of knowledge and adequate attitudes towards COVID-19 and its preventive measures after the SARS-CoV-2 pandemic. These were mainly stable, but with a long-term change in some attitudes in younger adults, those with a higher level of education, and individuals with no previous infection. These results should serve as a guide for health policy makers in decision-making to adopt effective response measures, implement targeted strategies for vulnerable groups, and ensure ongoing compliance in case of a new increase in the incidence of SARS-CoV-2 infections in the population.

## Figures and Tables

**Table 1:** Stratified analysis of long-term changes in the knowledge of COVID-19 disease and its preventive measures by age, educational level, high-risk conditions and previous infection.

Questions and Values	Age Group		Educational Level		High-Risk Conditions		Previous Infection	
	<60 Yrs	≥60 Yrs	Primary/Secondary	Higher	Yes	No	Yes	No
All people who become ill with COVID-19 will develop into a severe case								
Baseline, %	79.8	72.9	68.7	86.7	72.8	84.0	78.2	76.1
3-month follow-up, %	86.2	58.0	67.0	85.2	71.1	83.1	78.0	72.9
6-month follow-up, %	84.0	68.6	67.7	90.0	74.6	84.0	81.2	75.0
AD, % *	4.2	−4.3	−1.0	3.3	1.8	0.0	3.0	−1.1
*p*-value	0.400	0.577	0.879	0.486	0.764	1.000	0.600	0.861
Asymptomatic persons diagnosed with COVID-19 can transmit the infection								
Baseline, %	89.1	77.1	77.8	92.2	83.3	86.7	84.2	85.2
3-month follow-up, %	83.6	79.7	79.4	85.2	83.3	80.3	84.0	80.0
6-month follow-up, %	86.6	80.0	78.8	90.0	86.0	81.3	84.2	84.1
AD, % *	−2.5	2.9	1.0	−2.2	2.7	−5.4	0.0	−1.1
*p*-value	0.552	0.680	0.863	0.600	0.582	0.373	1.000	0.834
Persons diagnosed with COVID-19 can transmit infection despite vaccination								
Baseline, %	91.6	85.7	82.8	96.7	89.5	89.3	92.1	86.4
3-month follow-up, %	89.7	81.2	83.5	89.8	84.2	90.1	84.0	89.4
6-month follow-up, %	93.3	77.1	77.8	97.8	86.0	89.3	90.1	84.1
AD, % *	1.7	−8.6	−5.0	1.1	−3.5	0.0	−2.0	−2.3
*p*-value	0.624	0.193	0.372	0.650	0.420	1.000	0.621	0.671
The SARS-CoV-2 virus is spread by respiratory droplets from infected individuals when coughing/sneezing/talking/laughing/singing								
Baseline, %	92.4	88.6	89.9	92.2	91.2	90.7	91.1	90.9
3-month follow-up, %	91.4	89.9	86.6	95.5	91.2	90.1	95.0	85.9
6-month follow-up, %	95.0	94.3	92.9	96.7	93.9	96.0	96.0	93.2
AD, % *	2.6	5.7	3.0	4.5	2.7	5.3	4.9	2.3
*p*-value	0.424	0.228	0.447	0.193	0.449	0.191	0.152	0.577
Handwashing is important in reducing the risk of contracting COVID-19								
Baseline, %	91.6	91.4	92.9	90.0	93.9	88.0	90.1	93.2
3-month follow-up, %	90.5	92.8	92.8	89.8	91.2	91.5	90.0	92.9
6-month follow-up, %	92.4	87.1	90.9	90.0	89.5	92.0	89.1	92.0
AD, % *	0.8	−4.3	−2.0	0.0	−4.4	4.0	−1.0	−1.2
*p*-value	0.811	0.412	0.602	1.000	0.231	0.414	0.818	0.773
To prevent transmission of COVID-19, measures such as wearing face masks and avoiding crowds in enclosed spaces should be maintained								
Baseline, %	89.1	97.1	92.9	91.1	94.7	88.0	93.1	90.9
3-month follow-up, %	86.2	87.0	87.6	85.2	87.7	84.5	88.0	84.7
6-month follow-up, %	89.1	82.9	84.8	88.9	87.7	85.3	84.2	89.8
AD, % *	0.0	−14.2	−8.1	−2.2	−7.0	−2.7	−8.9	−1.1
*p*-value	1.000	0.005	0.070	0.619	0.061	0.631	0.046	0.779

* Abbreviations: AD; absolute difference (baseline—6-month follow-up).

**Table 2:** Stratified analysis of long-term changes in attitudes about COVID-19 disease and its preventive measures by age, educational level, high-risk conditions and previous infection.

Questions and Values	Age Group		Educational Level		High-Risk Conditions		Previous Infection	
	<60 yrs	≥60 yrs	Primary/Secondary	Higher	Yes	No	Yes	No
I consider myself susceptible to developing severe disease if I become ill with COVID-19								
Baseline, %	42.0	42.9	40.4	44.4	21.9	73.3	37.6	47.7
3-month follow-up, %	44.8	27.5	34.0	43.2	15.8	74.6	37.0	40.0
6-month follow-up, %	47.1	32.9	41.4	42.2	19.3	76.0	39.6	44.3
AD, % *	5.1	−10.0	1.0	−2.2	−2.6	2.7	2.0	−3.4
*p*-value	0.434	0.223	0.885	0.764	0.623	0.707	0.773	0.650
I consider that my inner circle has complied with the preventive measures to avoid becoming ill with COVID-19								
Baseline, %	76.5	84.3	90.9	66.7	78.9	80.0	74.3	85.2
3-month follow-up, %	80.2	91.3	92.8	75.0	86.0	81.7	80.0	89.4
6-month follow-up, %	76.5	92.9	93.9	70.0	84.2	80.0	76.2	89.8
AD, % *	0.0	8.6	3.0	3.3	5.3	0.0	1.9	4.6
*p*-value	1.000	0.111	0.420	0.631	0.307	1.000	0.744	0.362
I consider that my inner circle has complied with the vaccination recommendations given by the health authorities								
Baseline, %	88.2	94.3	92.9	87.8	90.4	90.7	89.1	92.0
3-month follow-up, %	84.5	88.4	89.7	81.8	83.3	90.1	86.0	85.9
6-month follow-up, %	79.8	90.0	91.9	74.4	83.3	84.0	80.2	87.5
AD, % *	−8.4	−4.3	−1.0	−13.4	−7.1	−6.7	−8.9	−4.5
*p*-value	0.077	0.346	0.788	0.022	0.117	0.220	0.079	0.320
I consider that it is better to develop immunity by getting sick with COVID-19 than by being vaccinated								
Baseline, %	45.4	58.6	54.5	45.6	50.9	49.3	48.5	52.3
3-month follow-up, %	51.7	65.2	62.9	50.0	61.4	49.3	55.0	58.8
6-month follow-up, %	52.9	50.0	53.5	50.0	50.0	54.7	50.5	53.4
AD, % *	7.5	−8.6	−1.0	4.4	−0.9	5.4	2.0	1.1
*p*-value	0.244	0.311	0.887	0.551	0.895	0.513	0.778	0.880
It is convenient for the general population to wear a face mask correctly (covering the nose and mouth) to prevent COVID-19 in crowded closed environments								
Baseline, %	92.4	91.4	92.9	91.1	93.0	90.7	90.1	94.3
3-month follow-up, %	87.9	79.7	86.6	83.0	85.1	84.5	85.0	84.7
6-month follow-up, %	75.6	75.7	78.8	72.2	74.6	77.3	70.3	81.8
AD, % *	−16.8	−15.7	−14.1	−18.9	−18.4	−13.4	−19.8	−12.5
*p*-value	<0.001	0.012	0.004	0.001	<0.001	0.026	<0.001	0.011
I consider that COVID-19 had a negative influence on my daily life								
Baseline, %	51.3	52.9	49.5	54.4	54.4	48.0	44.6	60.2
3-month follow-up, %	33.6	44.9	46.4	28.4	45.6	25.4	28.0	49.4
6-month follow-up, %	31.9	41.4	39.4	31.1	40.4	28.0	32.7	38.6
AD, % *	−19.4	−11.5	−10.1	−23.3	−14.0	−20.0	−11.9	−21.6
*p*-value	0.002	0.176	0.153	0.002	0.034	0.012	0.083	0.004

* Abbreviation: AD absolute difference (baseline—6-month follow-up).

## Data Availability

The data presented in this study are available on request from the corresponding author.

## References

[1] Munster V.J., Koopmans M., van Doremalen N., van Riel D., de Wit E. (2020). A novel coronavirus emerging in China—Key questions for impact assessment. N. Engl. J. Med..

[2] Lai C.C., Shih T.P., Ko W.C., Tang H.J., Hsueh P.R. (2020). Severe acute respiratory syndrome coronavirus 2 (SARS-CoV-2) and coronavirus disease-2019 (COVID-19): The epidemic and the challenges. Int. J. Antimicrob. Agents.

[3] Kaur S., Bherwani H., Gulia S., Vijay R., Kumar R. (2021). Understanding COVID-19 transmission, health impacts and mitigation: Timely social distancing is the key. Environ. Dev. Sustain..

[4] Liu Y., Morgenstern C., Kelly J., Lowe R. (2021). CMMID COVID-19 Working Group; Jit, M. The impact of non-pharmaceutical interventions on SARS-CoV-2 transmission across 130 countries and territories. BMC Med..

[5] Falcón-Romero M., Rodriguez-Blázquez C., Romay-Barja M., Forjaz M.J. (2023). Evolución de las preocupaciones, percepciones y actitudes de la población española ante la pandemia de COVID-19. Rev. Esp. Sociol..

[6] Martínez-Baz I., Miqueleiz A., Egüés N., Casado I., Burgui C., Echeverría A., Navascués A., Fernández-Huerta M., García Cenoz M., Trobajo-Sanmartín C. (2023). Effect of COVID-19 vaccination on the SARS-CoV-2 transmission among social and household close contacts: A cohort study. J. Infect. Public Health.

[7] Barbeito I., Precioso D., Sierra M.J., Vegas-Azcárate S., Fernández Balbuena S., Vitoriano B., Goméz-Ullate D., Cao R., Monge S. (2023). Study Group for Non-Pharmaceutical Interventions in Spain. Effectiveness of non-pharmaceutical interventions in nine fields of activity to decrease SARS-CoV-2 transmission (Spain, September 2020–May 2021). Front. Public Health.

[8] Rodríguez-Blázquez C., Romay-Barja M., Falcón M., Ayala A., Forjaz M.J. (2021). The COSMO-Spain survey: Three first rounds of the WHO behavioral insights tool. Front. Public Health.

[9] Betsch C., Wieler L.H., Habersaat K., COSMO Group (2020). Monitoring behavioural insights related to COVID-19. Lancet.

[10] Martínez-Baz I., Bullón-Vela V., Soldevila N., Torner N., Palma D., García Cenoz M., Pérez G., Burgui C., Castilla J., Godoy P. (2023). Assessment of knowledge and attitudes over time in postacute COVID-19 environments: Protocol for an epidemiological study. JMIR Res. Protoc..

[11] Bullón-Vela V., Toledo D., Echeverría A., Godoy P., Cenoz M.G., Parrón I., Castilla J., Domínguez A., Martínez-Baz I. (2024). Absence of short-term changes in knowledge and attitudes among household contacts of COVID-19 cases during the post-acute phase of the pandemic in Catalonia and Navarre, Spain. Front. Public Health.

[12] Instituto de Salud Carlos III Monitorización del Comportamiento y las Actitudes de la Población Relacionadas con la COVID-19 en España (COSMO-SPAIN): Estudio OMS. https://portalcne.isciii.es/cosmo-spain/.

[13] Orden SND/726/2023, de 4 de Julio, por la que se publica el Acuerdo del Consejo de Ministros de 4 de Julio de 2023, por el que se declara la finalización de la situación de crisis sanitaria ocasionada por la COVID-19. https://www.boe.es/boe/dias/2023/07/05/pdfs/BOE-A-2023-15552.pdf.

[14] Siddiquea B.N., Shetty A., Bhattacharya O., Afroz A., Billah B. (2021). Global epidemiology of COVID-19 knowledge, attitude and practice: A systematic review and meta-analysis. BMJ Open.

[15] Rahman M.M., Marzo R.R., Chowdhury S., Qalati S.A., Hasan M.N., Paul G.K., Abid K., Sheferaw W.E., Mariadass A., Chandran D. (2022). Knowledge, attitude and practices toward coronavirus disease (COVID-19) in Southeast and South Asia: A mixed study design approach. Front. Public Health.

[16] Gupta R.M., Mann S.K., Negi A. (2022). KAP (Knowledge, Attitude and Practices) analysis during two consecutive waves of COVID-19 in India. GJSFR.

[17] World Health Organization (WHO) (2020). Pandemic fatigue: Reinvigorating the public to prevent COVID-19: Policy framework for supporting pandemic prevention and management: Revised version November 2020. Copenhagen: WHO Regional Office for Europe.

[18] Agurto-Ramírez A., Pino-Rosón C., Ayala A., Falcón M., Rodríguez-Blázquez C., Forjaz M.J., Romay-Barja M. (2023). Association between pandemic fatigue and disease knowledge, attitudes, concerns, and vaccination intention at two key moments of the COVID-19 pandemic. Int. J. Public Health.

